# Spring thaw ionic pulses boost nutrient availability and microbial growth in entombed Antarctic Dry Valley cryoconite holes

**DOI:** 10.3389/fmicb.2014.00694

**Published:** 2014-12-11

**Authors:** Jon Telling, Alexandre M. Anesio, Martyn Tranter, Andrew G. Fountain, Thomas Nylen, Jon Hawkings, Virendra B. Singh, Preeti Kaur, Michaela Musilova, Jemma L. Wadham

**Affiliations:** ^1^School of Geographical Sciences, University of BristolBristol, UK; ^2^Department of Geology, Portland State UniversityPortland, OR, USA; ^3^School of Environmental Sciences, Jawaharlal Nehru UniversityNew Delhi, India; ^4^School of Chemistry, University of BristolBristol, UK

**Keywords:** Antarctica, cryoconite, ionic pulse, nitrogen fixation, photosynthesis, bacterial production, McMurdo Dry Valleys, microbial ecology

## Abstract

The seasonal melting of ice entombed cryoconite holes on McMurdo Dry Valley glaciers provides oases for life in the harsh environmental conditions of the polar desert where surface air temperatures only occasionally exceed 0°C during the Austral summer. Here we follow temporal changes in cryoconite hole biogeochemistry on Canada Glacier from fully frozen conditions through the initial stages of spring thaw toward fully melted holes. The cryoconite holes had a mean isolation age from the glacial drainage system of 3.4 years, with an increasing mass of aqueous nutrients (dissolved organic carbon, total nitrogen, total phosphorus) with longer isolation age. During the initial melt there was a mean nine times enrichment in dissolved chloride relative to mean concentrations of the initial frozen holes indicative of an ionic pulse, with similar mean nine times enrichments in nitrite, ammonium, and dissolved organic matter. Nitrate was enriched twelve times and dissolved organic nitrogen six times, suggesting net nitrification, while lower enrichments for dissolved organic phosphorus and phosphate were consistent with net microbial phosphorus uptake. Rates of bacterial production were significantly elevated during the ionic pulse, likely due to the increased nutrient availability. There was no concomitant increase in photosynthesis rates, with a net depletion of dissolved inorganic carbon suggesting inorganic carbon limitation. Potential nitrogen fixation was detected in fully melted holes where it could be an important source of nitrogen to support microbial growth, but not during the ionic pulse where nitrogen availability was higher. This study demonstrates that ionic pulses significantly alter the timing and magnitude of microbial activity within entombed cryoconite holes, and adds credence to hypotheses that ionic enrichments during freeze-thaw can elevate rates of microbial growth and activity in other icy habitats, such as ice veins and subglacial regelation zones.

## Introduction

The McMurdo Dry Valleys (MDV) are polar deserts comprising the largest ice-free expanse in Antarctica, and represent one of the coldest and driest ecosystems on Earth (Fountain et al., 1999). Glacial melt is critically important for supplying liquid water not only to supraglacial ecosystems (cryoconite holes, cryolakes, and supraglacial streams) (Mueller et al., 2001; Tranter et al., 2004; Foreman et al., 2007) but also downstream ecosystems (ephemeral streams, sandy soils, and perennially ice covered lakes) in a short 6–10 week melt season during the Austral summer (Fountain et al., 1999).

Surface temperatures on the glaciers rarely exceed 0°C even in mid-summer so that the melting of the glacier surface typically initiates through the warming of relatively low albedo surface sediment (cryoconite) (Fountain et al., 2008). Cryoconite melts its way down into the glacier to form cylindrical water filled holes, with the surface water refreezing to form ice-lids that isolate the holes from the atmosphere. Eventually the cryoconite holes reach an equilibrium depth where the rate of melt deepening supported by incoming insolation through the ice-lids matches the rate of surface ablation (Mcintyre, 1984; Fountain et al., 2008). Individual holes can be isolated from the atmosphere for a year up to a decade or more (Tranter et al., 2004; Fountain et al., 2008), with a combination of physical processes (ice melt, mineral dissolution, and mineral precipitation reactions) and microbial activities forming unique water chemistries distinct from those of the surrounding ice (Tranter et al., 2004).

The presence of liquid water, nutrients, and sunlight within the cryoconite holes supports a wide range of prokaryotic and eukaryotic microbial life during the Austral summer (Wharton et al., 1981; Christner et al., 2003; Porazinska et al., 2004; Foreman et al., 2007), with significant rates of potential photosynthesis (Bagshaw et al., 2011), bacterial production and enzymatic activity (Foreman et al., 2007). The disparity in the organic matter content of MDV valley floor soils (0.2 ± 0.04 mg C g^−1^; Barrett et al., 2007) and glacier cryoconite (mean of 1.0 mg C g^−1^ on Canada Glacier; Bagshaw et al., 2013) may be evidence that MDV cryoconite holes are net autotrophic ecosystems, although this has yet to be proven (Bagshaw et al., 2013). The ability of microbes on MDV glacier surfaces to fix nitrogen from the atmosphere has also yet to be tested, and could potentially provide an important source of bioavailable nitrogen for glacier microbial communities as previously documented on Arctic glaciers (Telling et al., 2011, 2012b). Ultimately, periodic warming events flush the cryoconite holes out, providing a potentially important supply of water and nutrients to downstream ephemeral streams and perennially ice covered lakes (Fountain et al., 2004; Foreman et al., 2007; Bagshaw et al., 2013).

One important aspect of cryoconite hole ecology that has not been explored to date is the impact of freeze-thaw events on microbial activity. Pulses of melt water during the first 2 weeks of the summer melt season (a significant fraction of the typical 6–10 week melt season) have highly elevated ionic concentrations up to two orders of magnitude greater than in fully thawed holes, with large diurnal variations (Fountain et al., 2008). These “ionic pulses” likely reflect the initial melting of brine ice formed during the freezing of cryoconite hole waters at the end of the previous melt season (Fountain et al., 2008). The impact of these ionic pulses on aqueous nutrient concentrations and microbial ecology within cryoconite holes has yet to be assessed. Analogous studies on early snow melt have documented the preferential elution of some ions over others (Davies et al., 1982; Cragin et al., 1996). During snow crystal growth, less soluble species such as SO^2−^_4_, Mg^2+^, and Ca^2+^ are preferentially excluded from the ice crystals to form brine surface layers (Cragin et al., 1996). These brines are the first to melt in the spring, with SO^2^_4_, Mg^2+^ and Ca^2+^ appearing sooner and in higher concentration in initial snow melt than more soluble ions such as Cl^−^ (Cragin et al., 1996). It has been hypothesized that in MDV ice lidded cryoconite holes, early melt season nutrient rich “ionic pulses” may stimulate microbial growth after the winter freeze (Fountain et al., 2008).

In this paper, we aim to test the hypothesis that early melt season ionic pulse events have a significant impact on rates of microbial growth and activity within ice entombed cryoconite holes. We follow the temporal changes in cryoconite water chemistry and microbial activity (potential photosynthesis, heterotrophic bacterial production, and potential nitrogen fixation) in cryoconite holes through the start of the melt season on Canada Glacier in the MDV. This study will increase our understanding of cryoconite hole biogeochemistry, and help to define the role of cryoconite holes in the depauperate ecosystem of the MDV.

## Materials and methods

### Sampling and field analysis of samples

The sample site was located on the surface of Canada Glacier, located within the 34 km long Taylor Valley of the MDV, Antarctica. Eight ice-lidded cryoconite holes were sampled at four different time points on 25th November, 28th November, 30th November, and 5th December 2011, hereafter termed *T* = 0, *T* + 3, *T* + 5, and *T* + 10 (with the number referring to days since initial sampling). Eight ice cores with no underlying cryoconite were also sampled on 25th November 2011. All cryoconite holes were completely frozen at *T* = 0, with increasing melt water at later time points. The same cryoconite holes were not sampled repeatedly; rather, eight different cryoconite holes were destructively sampled in the same local 10 m^2^ area of the glacier centered around the following coordinates: S 77°37.546′, E 162°57.620′. In all cases, samples were taken using a hand driven Kovacs ice corer. Prior to sampling, the width and length of the cryoconite holes (identifiable by the different crystal structure of the refrozen ice lids) were measured using a ruler (±0.5 cm).

After coring, ice cores and fully frozen cryoconite holes were transferred to large polyethylene bags for and transported to the field laboratory. In the laboratory, cores were melted in the laboratory and samples treated as below for water samples. In cryoconite holes where liquid water was present, the ice lid was removed and the water depth, sediment depth, and hole depth were measured with a ruler (±0.5 cm). Water was sampled using a 50 ml plastic syringe and transferred into 1L HDPE bottles for later ion and nutrient analysis and 65 ml glass BOD (Biological Oxygen Demand) bottles for dissolved inorganic carbon (DIC) and pH measurements later the same day. All HDPE bottles were rinsed three times with sample prior to filling. The BOD bottles were filled from the bottom using a length of Tygon® tubing attached to the syringe, and the bottle allowed to overflow 3× their volume prior to capping with no headspace. Cryoconite samples were collected with a stainless steel scoop into 50 ml Falcon tubes. Ice samples were collected in polyethylene bags, melted in the laboratory and the water treated as below for water samples.

DIC was measured in a field laboratory by acidification of the water samples and measurement of the CO_2_ evolved using a portable PP systems EGM-4 infrared CO_2_ meter, with a coefficient of variation of 1% (Telling et al., 2010). pH was measured using a Thermo-Orion pH meter and probe. Water for later ion and nutrient analysis was filtered through Whatman Puradisc® 0.45 μm inline filters into 30 ml HDPE bottles after washing three times with sample, and immediately frozen at −20°C. Water for δ^18^O-H_2_O analysis were filtered through Whatman Puradisc® 0.45 μm inline filters into 30 ml HDPE bottles, leaving no headspace.

### Melted ice and cryoconite water geochemical analyses

Dissolved anions (Cl^−^, NO^−^_3_, SO^2−^_4_) and cations (Na^+^, K^+^, Ca^2+^, Mg^2+^) were analyzed on a dual system Thermofisher Dionex 5000 capillary ion chromatograph with IonPac AS11-HC (0.4 × 250 mm) and CS16 (0.5 × 250 mm) columns at 30°C, using 32.2 mM KOH and 30 mM methanesulfonic acid (MSA) as eluents for anions and cations, respectively. Detection limits for Cl^−^, NO^−^_3_, and SO^2−^_4_ were 0.5, 0.6, and 0.2 μmoles L^−1^ respectively, with coefficients of variation of 0.8, 2.4, and 1.9% (*n* = 10). Detection limits for Na^+^, K^+^, Ca^2+^, Mg^2+^ were 2.7, 1.4, 1.0, and 0.1 μmoles L^−1^ respectively, with coefficients of variation of 1.8, 2.7, 1.9 and 1.9% (*n* = 10). Dissolved organic carbon (DOC) was analyzed on a Shimadzu TOC-V CSN analyzer. The detection limit was 10.1 μmoles L^−1^ with a coefficient of variation of 6.4% (*n* = 6). NO^−^_2_ and NH^+^_4_ were analyzed on a Bran + Luebbe Autoanalyzer 3, with detection limits of 0.09 and 0.4 μmoles L^−1^ respectively, and coefficients of variation of 1.5 and 9.2%, respectively. PO^3−^_4_, total dissolved phosphorus (TP_(aq)_) and total dissolved nitrogen (TN_(aq)_) were analyzed on a Lachat QuickChem 8500 Series 2 Flow Injector Analyzer using standard QuikChem® methods 31-115-01-1-I, 31-115-01-3-F and 31-107-04-3-A, respectively. Detection limits were 0.036, 1.4, and 1.0 μmoles L^−1^ for PO^3−^_4_, TP_(aq)_ and TN_(aq)_ respectively, with coefficients of variation of 0.5, 3.4, and 7.4% (*n* = 6). Dissolved organic nitrogen (DON) was defined as TN_(aq)_ − [NH^+^_4_ + NO^−^_2_ + NO^−^_3_]. Dissolved organic phosphorus (DOP) was defined as TP_(aq)_-PO^3−^_4_.

The δ^18^O-H_2_O of samples (a proxy for the percentage of ice melt within the cryoconite holes; see below) was analyzed using a Picarro L1120-i cavity ringdown spectrometer, with a precision of ±0.2‰. Inorganic carbon speciation in *T* + 10 cryoconite holes (the only time point where pH was reliably measured) was modeled using the computer program PHREEQ-C using its standard database.

### Cryoconite geochemical analyses

Three cryoconite samples from each time point were analyzed for TOC, IC, and TN. All eight cryoconite samples from each time point were analyzed for solid phase nitrogen and phosphorus speciation. Dried cryoconite samples (105°C, overnight) were analyzed for total organic carbon (TOC) and total nitrogen (TN) on a Eurovector EA3000 Elemental Analyzer. Detection limits for TOC and TN were 100 μg C g^−1^ and 100 μg N g^−1^ respectively, with coefficients of variation for duplicate analyses of 9.5 and 5.3%. Exchangeable NH^+^_4_, NO^−^_3_, and NO^−^_2_ in wet cryoconite were extracted using a 2M KCl method (Telling et al., 2011). Extracts were analyzed on a Bran and Luebbe Autoanalyzer 3. Results were converted to dry weights after weighing cryoconite before and after oven drying. The average coefficients of variation for duplicate NH^+^_4_ and NO^−^_3_ analyses were 35.7 and 37.8%, respectively. Potentially bioavailable and residual phosphorus (P_residual_) in dried cryoconite were analyzed using a three stage sequential digestion method using (1) 1 M MgCl_2_, (2) 0.1 M NaOH and (3) acid persulfate. Extracts were analyzed on a Shimadzu mini UV-vis spectrophotometer. The average coefficients of variation for duplicate cryoconite samples for the MgCl_2_, NaOH, and persulfate digestion steps were 10.1, 6.2, and 10.3%, respectively.

### Cryoconite microbiological analyses

Three cryoconite samples from each time point were analyzed for total cell counts, autofluorescent counts, and biomass estimates. All eight samples at each time point were assayed for potential photosynthesis, bacterial production, and potential nitrogen fixation. Total and autofluorescent cell counts were enumerated by epifluorescent microscopy (Stibal et al., 2012). After thawing, 20 mg of the cryoconite were placed into a pre-weighed sterile Eppendorf tube and 1 mL of pre-sterilized deionized water, gluteraldehyde (2% final concentration) and sodium pyrophosphate (0.001 M final concentration) were added and vortexed for 1 min. The mixture was then sonicated for 2 min, shaken vigorously and 100 μl of the supernatant diluted 1:10 with autoclaved Milli-Q water. Microbial cells were stained with 4′, 6-diamidino-2-phenylindole (DAPI, Sigma) (10 μg mL^−1^ final concentration) and samples filtered onto black 0.22 μm polycarbonate filters (Millipore). Over 300 stained bacterial cells per slide were counted in duplicate using epifluorescence microscopy, and procedural blanks (sterilized deionized water) were subtracted from the sample counts. The same procedure was followed to enumerate photoautotrophic microbes using chlorophyll autofluorescence instead of staining the samples with DAPI (Stibal et al., 2012). Individual cell dimensions were digitally measured and biovolume calculated for each sample using an F-view II CCD (Olympus) and Cell^∧^f software (Olympus) (Bellas et al., 2013). Conversion factors reported by Bellas et al. (2013) were used to calculate the carbon content (fg C cell^−1^) from biovolume.

Due to time constraints, rates of microbiological activity were measured on cryoconite samples with the addition of cryoconite water from the same hole, rather than analyzing water and cryoconite samples separately. A drawback of this approach is that the incubations had different cryoconite:water volumes to *in situ* holes. Previous research has demonstrated that microbiological activity is focused in cryoconite (e.g., bacterial production rates are on average 95% higher in cryoconite rather than overlying water on Canada Glacier; Foreman et al., 2007), hence the microbial rates of this study likely dominantly reflect rates in the cryoconite debris. Potential photosynthesis in cryoconite was measured using a modified ^14^C-NaHCO_3_ method (Telling et al., 2010), within the Lake Hoare radioactive laboratory. Samples of sediment were placed in 8 ml polyethylene tubes to give a similar sediment depth (0.5 cm) to the *in situ* cryoconite holes. Water from the respective cryoconite hole was then added to the vial to leave no headspace, then 2 μl of a stock solution of a 37 MBq ^14^C-NaHCO_3_ solution (Perkin Elmer) was added. For each sample triplicate light, triplicate dark (covered with aluminum foil), and triplicate killed (100 μl 50% v/v glutaraldehyde added) vials were incubated for 1.5 h in an ice bath under a lamp. The average photosynthetically active radiation (PAR) at the surface of the ice bath measured using an Apogee PAR sensor and meter was 100 μmoles m^−2^ s^−1^ with a spatial variation of ±20 μmoles m^−2^ s^−1^. It should be noted that the actual PAR levels below ice lids in the cryoconite holes were not measured hence these rates of photosynthesis should be viewed as potential only. Vials were incubated for 1.5 h then removed from the ice bath and filtered through 0.45 μm cellulose nitrate filters. The pH of the filtrate was immediately raised to >pH 10 by the addition of pellets of NaOH prior to disposal. The filters were fumigated with 50% glutaraldehyde and 37% v/v HCl for >1 h, and the filters placed in scintillation vials. Vials were transported to the main laboratory (McMurdo), where 10 ml of Cytoscint ES scintillation cocktail was added and DPM values measured using a Beckman LS6000 scintillation counter, calibrated using external quench standards. Rates of photosynthesis (μg C g^−1^ h^−1^) were calculated following the procedures of Telling et al. (2010).

Bacterial production was measured using ^3^H leucine uptake within the field laboratory (Anesio et al., 2010). Quadruplicate cryoconite samples were placed into sterile 1.8 ml microcentrifuge tubes and ^3^H-leucine added to give a final concentration of 100 nM. One of the four tubes was killed using the addition of 100 μl of 50% glutaraldehyde. Tubes were wrapped in aluminum foil, incubated in an ice bath for 1.5 h, then activity terminated by the addition of 100 μl 50% glutaraldehyde. Samples were then transported to the Crary Laboratory (McMurdo), where 90 μl of 100% ice cold trichloroacetic acid was added to samples, and the tubes centrifuged at 16,000 g for 10 min. The pellet was washed sequentially with 5% trichloracetic acid and 80% ethanol and samples dried and weighed. Finally, 1 ml of Cytosint ES cocktail was added to samples, and DPM counted on a Beckman LS6000 scintillation counter calibrated using external quench standards. Bacterial production rates (μg C g^−1^ h^−1^) were calculated following the methods of Anesio et al. (2010).

Potential nitrogen fixation was measured by a modified acetylene assay (Stewart et al., 1967; Telling et al., 2011). For each sample, triplicate 30 ml vials containing 0.5 cm deep sediment had 1.5 ml of acetylene added and incubated for 24 h in an ice bath under a 100 ± 20 μmoles cm^−2^ s^−1^ PAR fluorescent lamp. A further 30 ml bottle for each sample was incubated as above but without the addition of acetylene to check for *in situ* production of ethylene. A total of 13 bottles were run during the incubations with no sediment but with acetylene added to correct for the starting concentration of ethylene. After sampling, the cryoconite in each bottle was dried and weighed. Positive potential nitrogen fixation assays were defined as those bottles with an ethylene content more than 3 × SD of the acetylene only blanks after the mean blank value had been subtracted. Ethylene results in ppm were converted to μmoles after accounting for ethylene dissolution in water (Breitbarth et al., 2004). Rates were normalized to units of μg N g^−1^ assuming a 3:1 ratio between ethylene generated and dinitrogen fixed (Stewart et al., 1967) and a linear production of ethylene over time (Telling et al., 2011).

### Numerical analysis

#### Enrichment factors

Enrichment factors, where for example ion and nutrient concentrations in cryoconite holes are normalized to mean concentrations in the surrounding ice, can greatly facilitate the identification of biogeochemical sources and sinks (Tranter et al., 2004). In this study we define an enrichment factor (*XF*_0−*ice*_) between fully frozen cryoconite holes and surrounding ice cores as follows:

$$XF_{T0-ice}=\frac{C_{T=0}}{mean\;C_{ice}}$$

where *C*_*T* = 0_ is the concentration in the fully frozen cryoconite hole (*T* = 0), and mean *C*_*ice*_ is the mean concentration in the ice cores.

Similarly, we define enrichment factors between partially melted cryoconite holes (*T* + 3, *T* + 5, *T* + 10) and fully frozen cryoconite holes (*T* = 0) to facilitate the identification of biogeochemical processes occurring during early melt freeze-thaw effects as follows (using the example for *T* + *n*):

$$XF_{Tn-T0}=\frac{C_{T+n}}{mean\;C_{T=0}}$$

where *C*_*T* + *n*_ is the concentration, nutrient ratio or microbial rate in *T* + 3, *T* + 5, or *T* + 10 cryoconite holes and mean *C*_*T* = 0_ frozen is the mean concentration or microbial rate in the fully frozen cryoconite holes (*T* = 0).

#### Correlations against δ^18^O-H_2_O, a proxy for the percentage ice melt

The percentage ice melt in individual cryoconite holes at each time point is likely to vary substantially due to differential shading effects from variations in local topography and hole dimensions (Table 1). We therefore use the δ^18^O of the water as a more quantitative proxy than time for the percentage of ice melt within each individual hole, since the initial melt water should be enriched in the lighter ^16^O-H_2_O. ^16^O-H_2_O preferentially partitions into the water phase relative to the heavier ^18^O-H_2_0 (Hoefs, 1980). We use Pearson correlation coefficients (SPSS statistical software package) to test the significance of relationships between δ^18^O-H_2_0 and aqueous ions and δ^18^O-H_2_0 and cryoconite microbial activity. We transformed the data by taking the logarithm of aqueous ion and cryoconite activity data to linearise the data prior to carrying out the Pearson correlation analyses.

**Table 1:** **Physical data for cryoconite holes, Canada Glacier**.

**Time**	**Date (m/d/y)**	**Hole diameter (cm)**	**Hole depth (cm)**	**Sediment depth (cm)**	**Water depth (cm)**	**Ice lid depth (cm)**	**Isolation age (years)**
*T* = 0	11.25.11	25.8±5.6	16.2±5.5	1.5±0.5	0.0±0.0	16.2±5.5	4.2±4.2
*T* + 3	11.28.11	47.3±30.4	15.8±4.1	0.8±0.3	1.0±1.0	12.9±4.5	N/A
*T* + 5	11.30.11	46.9±9.6	14.5±4.9	2.5±1.1	4.8±0.8	10.8±7.9	N/A
*T* + 10	12.05.11	43.0±12.7	25.2±4.7	0.6±0.3	12.1±4.9	9.9±4.6	2.7±2.9

*Uncertainty is 1σ (n = 8)*.

#### Isolation age

The isolation age of the cryoconite waters from the surrounding supraglacial water system was estimated using chloride mass balance, following the approach of Fountain et al. (2004). Chloride is a conservative ion that will accumulate within ice lidded cryoconite holes as the holes melt into the glacier to keep pace with annual sublimation. We calculate the isolation ages of the frozen cryoconite holes (*T* = 0) using Equation 3 from Fountain et al. (2004), assuming: (a) an annual rate of sublimation of 8 cm a^−1^, (b) that the initial cryoconite water and additional annual ice melt have chloride concentrations equivalent to the mean chloride concentration of sediment free ice and, (c) that the ice lids contain no chloride (Fountain et al., 2004). In reality, there is likely to be some chloride contained within the ice lid, although at a smaller concentration than in the cryoconite water (Bagshaw et al., 2007). We make an estimate of the possible errors in our estimates of isolation age by calculating the potential mass of chloride in each of the cryoconite hole ice lids by assuming a mean ratio of chloride in ice lid:water of 1:3.6 (Bagshaw et al., 2007). For *T* + 10 cryoconite holes, where the volume of water exceeds that of the ice lids, the mean potential error in isolation ages is 28%. At *T* + 3 and *T* + 5, the volume of overlying ice lid greatly exceeded that of the water volume in the hole, which together with the uncertainties in the depth measurements of the low volumes of water precludes an accurate assessment of total chloride mass in each cryoconite hole and hence an accurate estimate of isolation age. The significance of relationships between isolation age and the mass of dissolved nutrients in each hole is assessed using linear regression analysis (SPSS statistical software package) after normalizing ion and nutrient data to the surface area of the holes in order to negate the impact of concentration and dilution of nutrients by freeze-thaw.

#### Aqueous nutrient mass balance within cryoconite holes

Comparison of the rate of influx of nutrients from ice ablation into cryoconite holes with rates of microbial growth can give insight into potential microbial nutrient limitations (Hodson et al., 2013). Here we estimate fluxes of dissolved nutrients from ice ablation into the *T* = 0 and *T* + 10 cryoconite holes with the measured nutrient demands of bacterial production and photosynthesis:

$$\begin{array}{l} F=C_{ice}\times Z\\ D=\frac{activity\;rate\;x\;M\;x\;R}{A} \end{array}$$

where F is the nutrient flux [DOC, DIC, TN_(aq)_ or TP_(aq)_] from ice ablation into the hole (μmoles m^−2^ h^−1^), based on the concentration of nutrients in the ice, *C*_ice_, and the ablation rate, *Z* (calculated assuming a constant ablation rate of 8 cm over 10 weeks, equivalent to 0.048 mm h^−1^), *D* is the estimated nutrient demand (C, N, or P) from bacterial production or photosynthesis within the cryoconite hole (μmoles m^−2^ h^−1^), activity rate is the measured rate of bacterial production or photosynthesis in the cryconite (μmoles g^−1^ h^−1^), *M* is the dry mass of cryoconite within the hole (calculated as the product of surface area, mean cryoconite depth, and mean cryoconite density (1.3 g ml^−1^; Section Physical Dimensions of Cryoconite Holes), the Redfield coefficient, *R*, is 1 for *C*, 1/16 for N, and 1/106 for P, and *A* is the plan view area of the cryoconite hole (m^2^).

The potential importance of nitrogen fixation for sustaining microbial growth within the fully melted cryoconite holes is estimated as follows. First, we convert the potential nitrogen fixation rates in units of μmoles N g^−1^ (**Table 6**) to potential carbon fixed (μmoles C g^−1^) assuming a C:N molar ratio of 6.6:1 (Redfield et al., 1963). We then compare these rates to the measured rates of bacterial production and photosynthesis.

## Results

### Physical dimensions of cryoconite holes

Mean water depths in the holes increased with time to a maximum of 12.1 ± 4.9 cm at *T* + 10 (Table 1). Mean cryoconite debris thicknesses ranged from 0.6 to 2.5 cm, with no consistent trend with time (Table 1). The mean cryoconite density (based on dry weights of known volumes of debris) was 1.3 ± 0.6 g dry cryoconite ml^−1^ (*n* = 64).

### Ice and aqueous geochemistry

**Figure 1:**
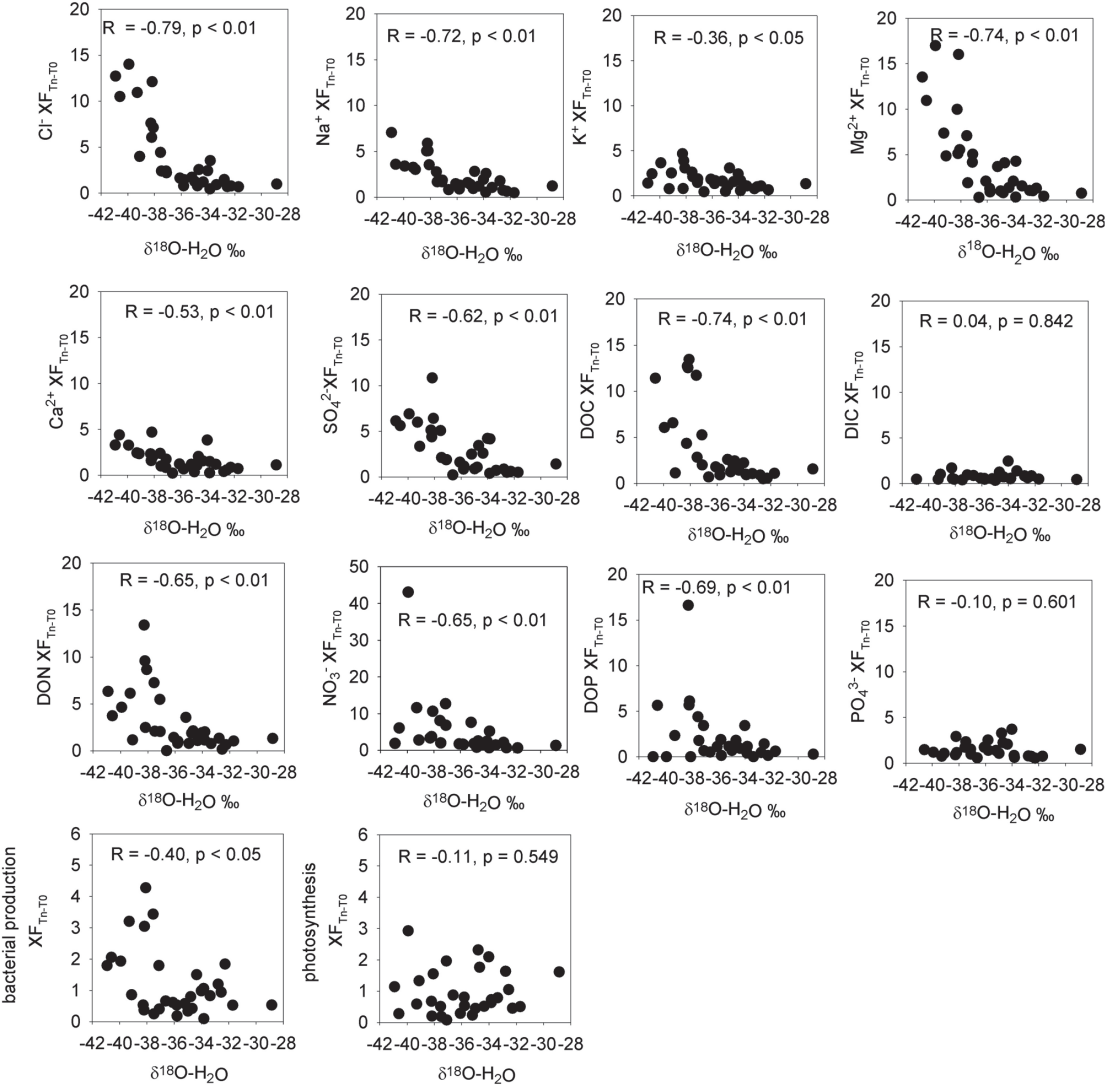
**Enrichment factors (XF) for aqueous ions and cryoconite bacterial production and photosynthesis vs. δ^18^O-H_2_0 (a measure of the % ice melt)**. Enrichment factors are the ratio of the measured aqueous concentrations of the ion to the mean concentration of that ion in initial frozen cryoconite holes. Statistics refer to Pearson correlation coefficients after taking the logarithm of the enrichment factor.

Ice core and aqueous geochemistry of cryoconite holes is summarized in Table 2. Mean chloride concentrations in fully frozen cryoconite holes were enriched 3.2× over ice cores (Table 3, Figure 1). Mean enrichment factors for Ca^2+^ (20.7×), Mg^2+^ (8.2×), and SO^2−^_4_ (9.1×) were higher than those of Cl^−^ (Table 3, Figure 1). Mean enrichment factors for DIC (3.1×) were similar to those of Cl^−^, DOC (2.4×), Na^+^ (2.0×), and K^+^ (2.6×). All dissolved nitrogen and phosphorus species (NO^−^_3_, NH^+^_4_, NO^−^_2_, DON, DOP, PO^3−^_4_) were depleted relative to Cl^−^ with enrichment factors <2× (Table 3, Figure 1).

**Table 2:** **Aqueous geochemistry of Canada Glacier ice cores and cryoconite holes**.

	**‰**	**μmoles L^−1^**													
	**δ^18^O-H_2_O**	**Cl^−^**	**DIC**	**Na^+^**	**K^+^**	**Ca^2^+**	**Mg^2^+**	**SO^2^−_4_**	**NO^−^_3_**	**NH^+^_4_**	**NO^−^_2_**	**DON**	**DOP**	**PO^3^−_4_**	**DOC**
Ice	−33.3 ± 2.7	9.3 ± 1.6	79 ± 18	12.4 ± 2.0	2.3 ± 1.2	4.0 ± 1.6	1.0 ± 0.3	1.0 ± 0.5	1.3 ± 0.7	0.04 ± 0.13	0.17 ± 0.18	2.1 ± 1.0	0.03 ± 0.01	0.06 ± 0.04	12.3 ± 12.2
*T* = 0	−33.4 ± 1.5	26.7 ± 19.2	242 ± 159	24.4 ± 13.7	5.9 ± 3.7	82.7 ± 98	5.9 ± 3.7	6.0 ± 7.8	1.6 ± 1.5	0.0 ± 0.0	0.05 ± 0.08	2.7 ± 1.5	0.06 ± 0.06	0.07 ± 0.07	29.3 ± 15.9
*T* + 3	−39.0 ± 1.4	275 ± 124	112 ± 1.4	92.5 ± 39.6	14.3 ± 6.6	241 ± 104	59.6 ± 28.6	36.4 ± 14.7	19.5 ± 20.8	1.8 ± 1.5	0.49 ± 0.47	14.9 ± 5.3	0.25 ± 0.09	0.09 ± 0.02	280 ± 101
*T* + 5	−35.9 ± 1.6	108 ± 64.6	190 ± 104	70.5 ± 41.2	12.6 ± 6.7	133 ± 41.7	27.0 ± 14.7	18.7 ± 7.8	6.0 ± 4.0	0.3 ± 0.6	0.15 ± 0.17	11.8 ± 12.2	0.36 ± 0.47	0.1 ± 0.05	103 ± 113
*T* + 10	−34.2 ± 2.4	36.3 ± 17.4	145 ± 70.6	32.6 ± 15.3	8.0 ± 5.0	81.4 ± 47.7	8.2 ± 6.5	9.1 ± 5.8	2.4 ± 0.9	0.0 ± 0.0	0.0 ± 0.0	3.5 ± 1.3	0.05 ± 0.04	0.12 ± 0.05	44.3 ± 13.7

*Uncertainty is 1σ (n = 8)*.

**Table 3:** **Mean enrichment factors for (a) Frozen cryoconite hole ions (*T* = 0) relative to mean ice core ions (*XF*_*T*0−*ice*_) and, (b) Partially melted cryoconite hole aqueous ions (*T* = 3, *T* = 5, *T* = 10) relative to mean starting frozen cryoconite hole ions (*XF*_*Tn*−*T*0_)**.

**Mean**	**δ^18^0-H_2_0**	**Cl^−^**	**DIC**	**Na^+^**	**K^+^**	**Ca^2^+**	**Mg^2^+**	**SO^2^−_4_**	**NO^−^_3_**	**NH^+^_4_**	**NO^−^_2_**	**DON**	**DOP**	**PO^3^−_4_**	**DOC**
*XF_T0-ice_*	0.99	3.2	3.1	2.0	2.6	20.7	5.7	6.1	1.6	0.8	0.8	1.3	1.8	1.4	2.4
*XF_T3-T0_*	1.18	9.3	0.5	3.8	2.4	2.9	10.2	6.1	12.2	9.7	9.7	5.6	4.4	1.1	9.6
*XF_T5-T0_*	1.09	3.6	0.8	2.9	2.1	1.6	4.6	3.1	3.7	3.0	3.0	4.4	6.2	1.5	3.5
*XF_T10-T0_*	0.91	1.2	0.6	1.3	1.5	1.0	1.4	1.5	1.5	0.0	0.0	1.3	0.9	1.9	1.5

**Figure 2:**
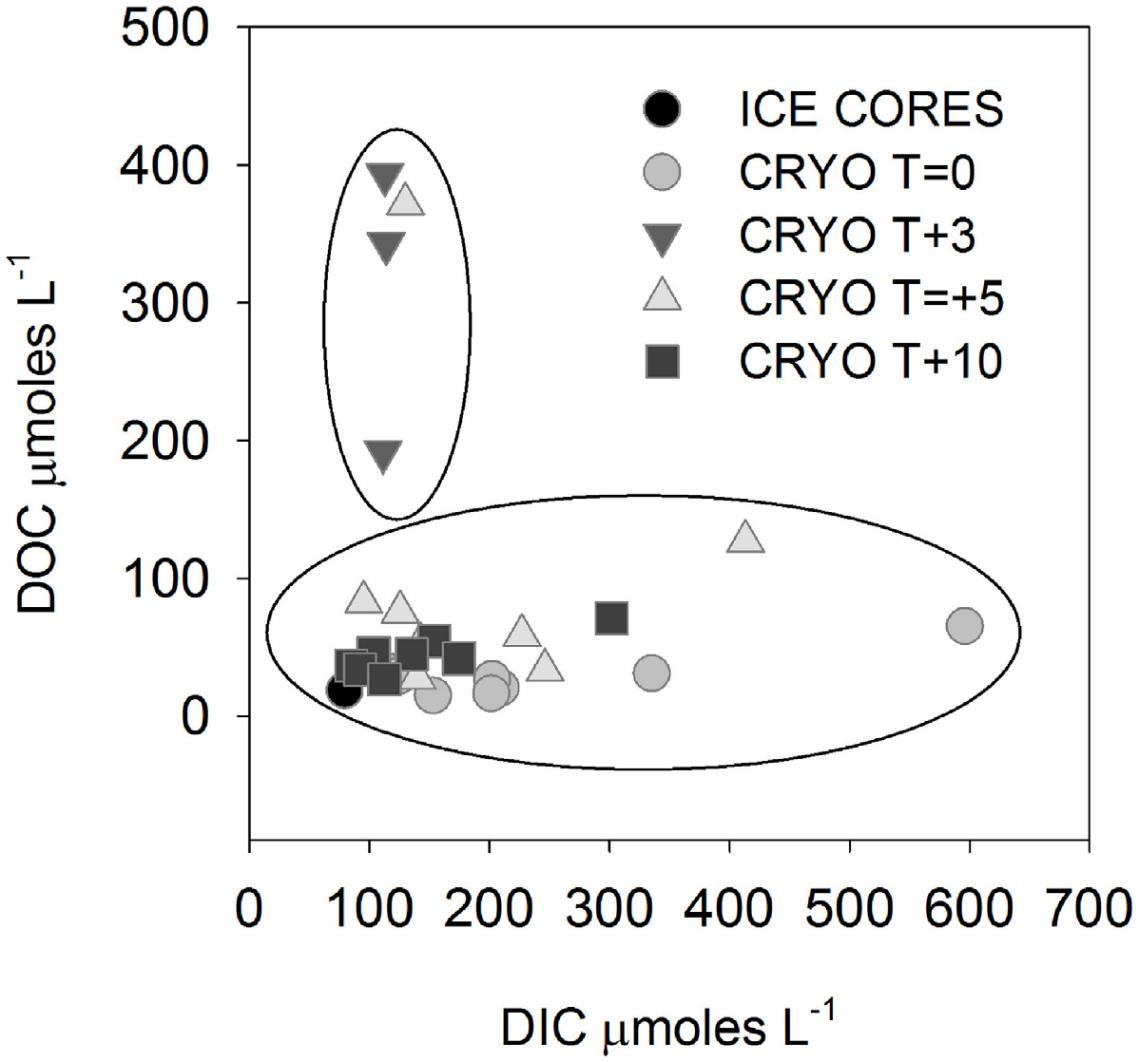
**Dissolved inorganic carbon (DIC) vs. dissolved organic carbon (DOC) in cryoconite hole waters**. Note that all *T* + 3 and one *T* + 5 samples are outliers to the remainder of the samples.

The isotopic values of δ^18^O-H_2_O during the first melt (*T* + 3) were lower relative to frozen cryoconite holes at *T* = 0 (Table 2). The mean Cl^−^ concentration in early melt waters (*T* + 3) was enriched 9.3× over fully frozen cryoconite holes (*T* = 0), alongside >9× increases in mean NO^−^_3_, NO^−^_2_, Mg^2+^, and DOC concentrations (Table 3, Figure 1). Enrichment factors for SO^2−^_4_, Na^+^, K^+^, Ca^2+^, DON, and DOP were all lower than that of Cl^−^, ranging from 6.1× to 2.4×. The mean enrichment factor for PO^3−^_4_ was close to unity (1.1×), while the mean DIC was depleted at *T* + 3 (0.5×) relative to *T* = 0. A plot of DIC against DOC indicates two distinct groups of samples: those with a relatively constant DOC level but varying DIC concentration (all of *T* = 0, all *T* + 10, all but one of *T* + 5; Pearson correlation, *R* = 0.395, *p* = 0.062, *n* = 23) and those with a relatively constant DIC concentration and higher and varying DOC concentrations (*T* + 3, one of *T* + 5; Figure 2).

There were significant positive Pearson correlations (*p* < 0.01) between δ^18^O-H_2_O and log enrichment factors for Cl^−^, SO^2−^_4_, NO^−^_3_, NO^−^_2_, TN, Mg^2+^, Ca^2+^, DON, DOP, and DOC and a less strong (*p* < 0.05) correlation between δ^18^O-H_2_0 and the log enrichment factor for K^+^ (Figure 1). There were no significant Pearson correlations between δ^18^O-H_2_0 and log enrichment factor for PO^3−^_4_ (*R* = −0.1, *p* = 0.601) or between δ^18^O-H_2_0 and log enrichment factor for DIC (*R* = 0.04, *p* = 0.842) (Figure 1).

pH was only measured in cryoconite holes at *T* + 10, with a mean of 8.9 ± 1.0. The dominant DIC species at *T* = was HCO^−^_3_, comprising 85–98% of inorganic carbon. CO_2(aq)_ concentrations varied from 0.05 to 12.9 μmoles L^−1^, with a mean of 2.6 ± 4.7 μmoles L^−1^.

**Figure 3:**
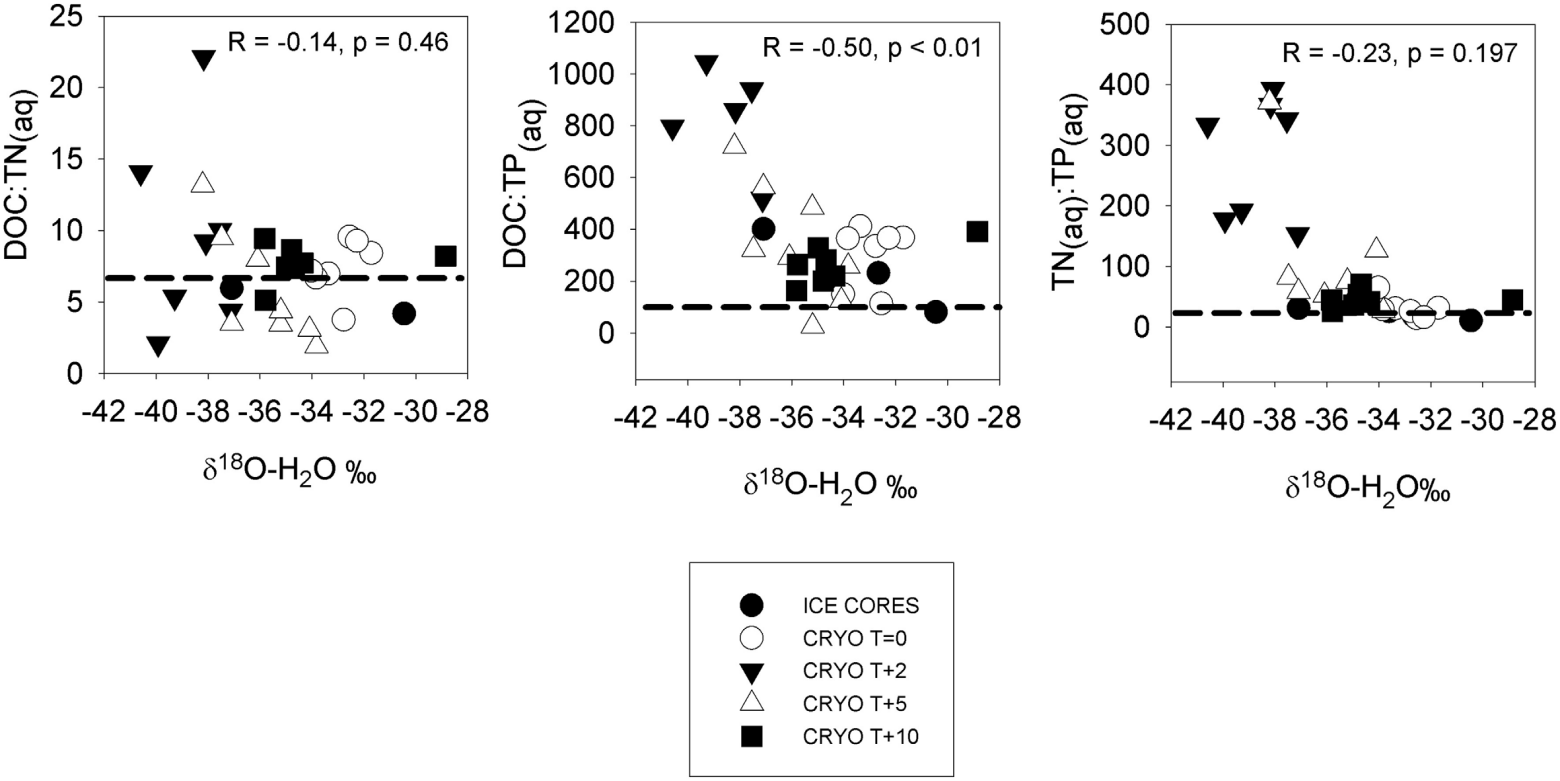
**Dissolved nutrient ratios in ice cores and cryoconite hole waters vs. δ^18^O-H_2_O (a measure of the % ice melt)**. Dotted horizontal lines show the Redfield ratios. Statistics refer to Pearson correlation coefficients.

The mean molar dissolved DOC:TN_(aq)_ in ice cores was 4.5 ± 1.3 (Table 4), lower than the Redfield ratio of 6.6. The mean DOC:TN_(aq)_ of cryoconite holes were elevated relative to the surrounding ice, bracketing the Redfield ratio at 5.9–9.6 (Table 4). There was a significant Pearson correlation (*p* < 0.01) between δ^18^O-H_2_O and DOC:TP_(aq)_ but not between δ^18^O-H_2_O vs. DOC:TN or δ^18^O-H_2_0 vs. TN:TP_(aq)_ (*R* = −0.14, *p* = 0.46, and *R* = −0.23, *p* = 0.197, respectively) (Figure 3).

**Table 4:** **Dissolved nutrient ratios in ice cores and cryoconite holes**.

	**C:N**	**C:P**	**N:P**	**DON:DIN**	**DOP:PO^3^−_4_**
Ice cores	4.5±1.3	218±137	46.8±24.9	1.9±1.2	0.6±0.2
*T* = 0	7.4±2.0	299±110	45.4±25.7	1.4±0.9	0.7±0.5
*T* + 3	9.6±6.8	831±199	101±62	1.2±1.2	2.9±1.1
*T* + 5	5.9±3.9	348±230	76.1±59.6	2.8±3.5	4.9±7.3
*T* + 10	7.6±1.3	268±73	36.8±12.4	1.9±1.3	0.4±0.2

*Uncertainty is 1σ (n = 8)*.

### Cryoconite chemistry and microbial activity

#### Solid phase composition

The mean organic and inorganic carbon concentrations of cryoconite were 0.9 ± 0.4 and 0.5 ± 0.3 mg C g^−1^, respectively (Table 5). Only three of the 12 samples had detectable total nitrogen (0.6 mg N g^−1^ at *T* = 0, and 0.5 mg N g^−1^ at *T* + 3, 0.4 mg N g^−1^ at *T* + 10), the remainder contained <0.1 mg N g^−1^ (Table 5).

**Table 5:** **Cryoconite solid phase nutrients**.

	**TOC**	**IC**	**TN**	**NH^+^_4__KCl_**	**NO^−^_3__KCl_**	**P_MgCl2_**	**P_NaOH_**	**P_residual_**
	**mg C g^−1^**	**mg C g^−1^**	**mg N g^−1^**	**ng N g^−1^**	**ng N g^−1^**	**μg P g^−1^**	**μg P g^−1^**	**μg P g^−1^**
*T* = 0	1.2±0.4	0.8±0.4	Detected	85±101	BDL	0.9±1.2	8.3±1.4	1403±330
*T* + 3	0.7±0.3	0.6±0.4	Detected	962±614	31±29	0.8±2.0	12.2±5.8	1409±346
*T* + 5	0.9±0.6	0.3±0.1	BDL	487±404	46±32	BDL	10.9±2.3	1516±109
*T* + 10	0.9±0.4	0.5±0.3	Detected	316±274	3.1±8.4	0.3±0.7	10.0±1.5	1493±70
Mean	0.9±0.4	0.5±0.3		451±490	20±29	0.5±1.2	10.3±3.4	1455±241

*Uncertainty is 1σ (n = 3 for TOC, IC and TN, n = 8 for remainder)*.

The mean concentrations of N_KCl_ varied with time. The lowest mean concentrations of NH^+^_4__KCl_ were at *T* = 0 (85 ng N g^−1^), with a maximum at *T* + 3 (962 ng N g^−1^) and reduced concentrations through *T* + 5 to a mean of 316 ng N g^−1^ at *T* + 10 (Table 5). NO^−^_3__KCl_ was below detection in all holes at *T* = 0, with higher concentrations at *T* + 3 and *T* + 5 (means of 31 and 46 ng N g^−1^), which fell to a mean of 3.1 ng N g^−1^ at *T* + 10 (Table 5).

The concentrations of P in cryoconite showed little variation with time of sampling (Table 5). The majority of P was in the form of P_residual_ (mean of 1460 μg P g^−1^), with low concentrations of P_MgCl2_ (mean of 0.5 μg P g^−1^) and P_NaOH_ (mean of 10.3 μg P g^−1^) (Table 5).

#### Microbial cell counts and activity measurements

The mean total microbial cell counts in cryoconite was 7.03 × 10^8^ ± 1.39 × 10^8^ cells wet g^−1^ sediment (all time points, *n* = 12; Table 6). The mean % of autotrophic cells (all time points, *n* = 12) was 10.6 ± 6.8% (Table 6). The mean total cell volume was 0.083 ± 0.04 μm^3^, while the mean autofluorescent cell volume was 3.9× larger at 0.32 ± 0.42 μm^3^ (Table 6). The mean DAPI cell biomass was 12.8 ± 7.0 μg C g^−1^ wet sediment, and mean autofluorescent cell biomass lower at 6.8 ± 12.3 μg C g^−1^ (Table 6). The mean autofluorescent cell biomass was higher at *T* + 3 (14.4 μg C g^−1^) relative to other time points (0.9–6.5 μg C g^−1^ wet sediment) (Table 6).

**Table 6:** **Cryoconite cells counts and biomass estimates**.

	**DAPI (total cell) counts Cells g^−1^ wet**	**Autoflourescent (AF) cell counts g^−1^ wet**	**DAPI volume μm^3^**	**AF volume μm^3^**	**% AF counts**	**DAPI biomass μg C g^−1^ wet**	**AF biomass μg C g^−1^ wet**
*T* = 0	8.0 × 10^8^±2.0 × 10^8^	5.0 × 10^7^±3.1 × 10^7^	0.11±0.03	0.07±0.04	5.8±2.9	19.6±5.4	0.9±0.8
*T* + 3	5.9 × 10^8^±1.2 × 10^8^	8.2 × 10^7^±4.1 × 10^7^	0.06±0.04	0.61±0.65	15.0±9.1	7.6±4.2	14.4±20.3
*T* + 5	6.8 × 10^8^±0.76 × 10^8^	4.5 × 10^7^±0.65 × 10^7^	0.05±0.01	0.17±0.14	6.7±0.2	6.9±1.3	1.6±1.1
*T* + 10	7.0 × 10^8^±2.7 × 10^8^	8.8 × 10^7^±3.9 × 10^7^	0.08±0.04	0.32±0.16	1.2±5.5	16.5±6.0	6.4±3.1
Mean	7.0 × 10^8^±1.4 × 10^8^	7.0 × 10^7^±3.5 × 10^7^	0.08±0.04	0.32±0.42	10.6±6.8	12.8±7.0	6.8±12.3

*Uncertainty is 1σ (n = 3)*.

**Figure 4:**
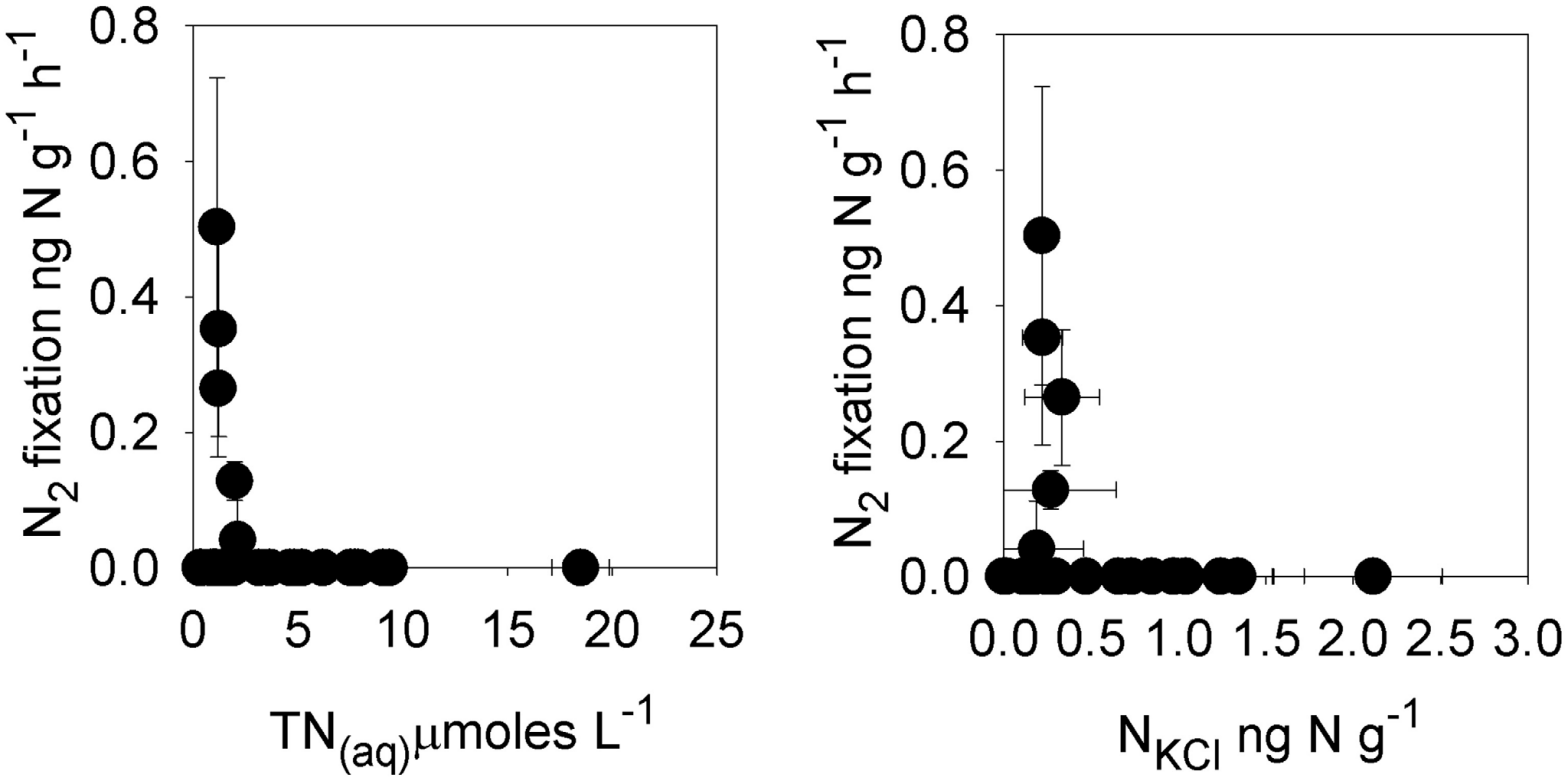
**Rates of potential nitrogen fixation in cryoconite vs. total dissolved nitrogen (TN_(aq)_) and cryoconite bound exchangeable nitrogen (N_KCl_)**.

Rates of bacterial production in individual cryoconite holes ranged from 0.3 to 6.8 ng C^−1^ g^−1^ h^−1^, with a mean of 2.4 ± 1.6 ng C^−1^ g^−1^ h^−1^ (*n* = 32; Table 7). Rates of potential photosynthesis in individual cryoconite holes ranged from 1.2 to 47.8 ng C^−1^ g^−1^ h^−1^, with a mean of 15.3 ± 11.7 ng C^−1^ g^−1^ h^−1^ (all time points, *n* = 32; Table 7). Potential nitrogen fixation was detected at *T* = 0 (one of eight samples, 0.13 ng N g^−1^ h^−1^) and *T* + 10 (four of eight samples, ranging from 0.04 to 0.50 ng N g^−1^ h^−1^) (Table 7). No potential nitrogen fixation was detected at *T* + 3 and *T* + 5 (Table 6). Potential nitrogen fixation was only detected when TN_(aq)_ was ≤9μmoles L^−1^, and TN_KCl_ was ≤330 ng N g^−1^ (Figure 4).

**Table 7:** **Rates of microbial activity in cryoconite**.

	**Bacterial production**	**Photosynthesis**	**Nitrogen fixation**	**Nitrogen fixation**
	**ng C g^−1^ h^−1^**	**ng C g^−1^ h^−1^**	**ng N g^−1^ h^−1^**	**μmoles C_2_H_4_ m^−2^ d^−1^**
*T* = 0	3.7±1.5	16.3±9.4	0.02±0.05	<1–2.3
*T* + 3	10.0±3.4	18.6±15.6	BDL	<1
*T* + 5	1.7±0.9	8.1±7.2	BDL	<1
*T* + 10	2.0±1.6	17.3±11.9	0.15±0.20	<1–4.9

*Uncertainty is 1σ (n = 8)*.

**Figure 5:**
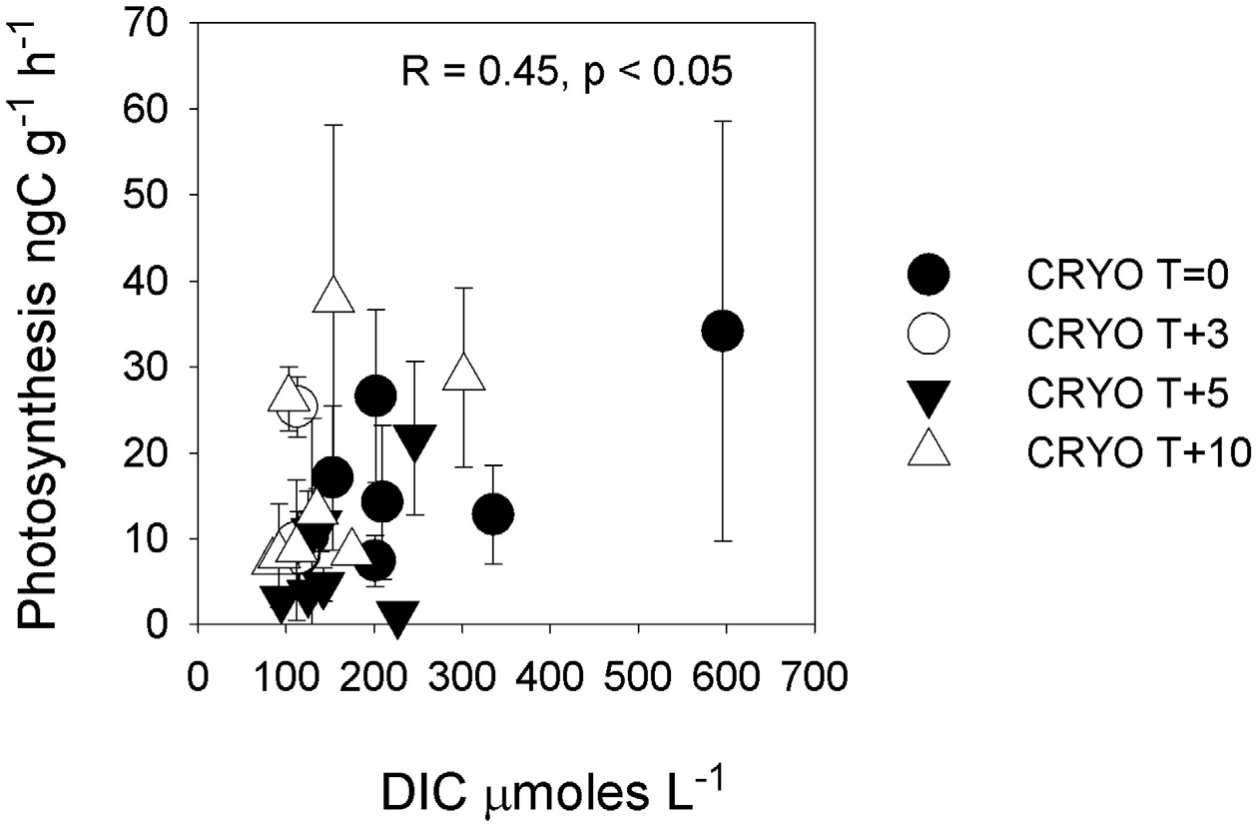
**Rate of photosynthesis in cryoconite vs. dissolved inorganic carbon (DIC)**. Statistics refer to Pearson correlation. Note that the significance of the relationship disappears when the highest DIC sample is removed.

There was no significant (*p* < 0.05) correlation between bacterial production and potential photosynthesis (*R* = 0.108, *p* = 0.562, *n* = 32, not shown). There was a significant correlation of bacterial production with δ^18^O-H_2_O (*R* = −0.40, *p* < 0.05, *n* = 32) but not between potential photosynthesis and δ^18^O-H_2_O (*R* = −0.11, *p* = 0.549, *n* = 32) (Figure 1). There was a weak but significant correlation between DIC and photosynthesis (Pearson correlation, *R* = 0.45, *p* < 0.05; Figure 5).

### Biogeochemistry as a function of isolation age

**Figure 6:**
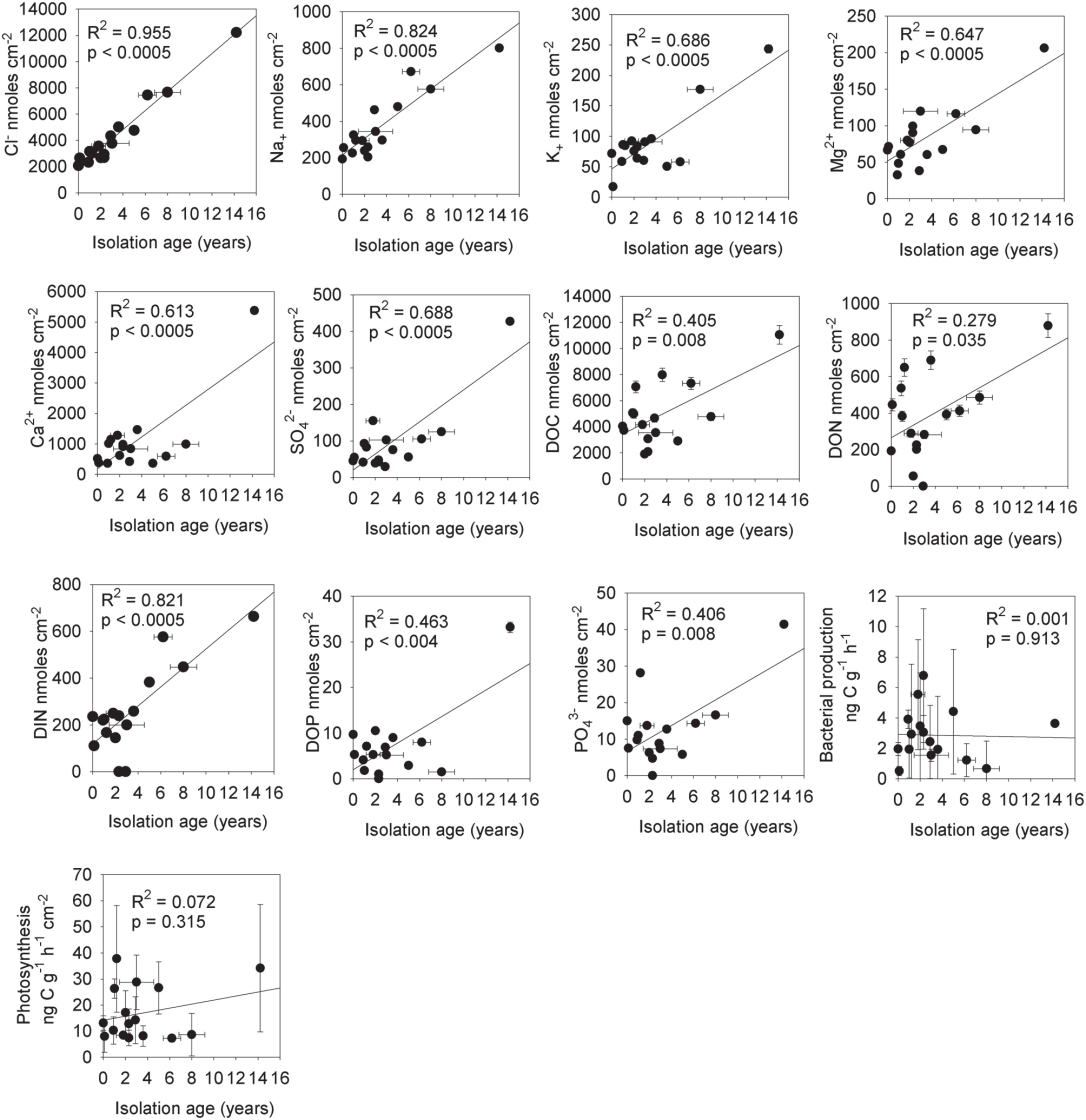
**The total mass of aqueous nutrients in cryoconite holes vs. their isolation age from the supraglacial hydrological system (based on chloride mass balance)**. Statistics are for linear regression analysis, *n* = 16 (*T* = 0 and *T* + 10).

The estimated mean isolation age (from the supraglacial water system) of the cryoconite holes (*T* = 0 and *T* + 10) from chloride mass balance was 3.4 ± 3.6 (1σ) years (Table 1). There were significant increases in the mass of solute per unit area for Cl^−^ (*R*^2^ = 0.955, *p* < 0.0005, *n* = 16), Na^+^ (*R*^2^ = 0.824, *p* < 0.0005, *n* = 16), K^+^ (*R*^2^ = 0.686, *p* < 0.0005), Mg^2+^ (*R*^2^ = 0.647, *p* < 0.0005, *n* = 32), Ca^2+^ (*R*^2^ = 0.613, *p* < 0.0005, *n* = 16) and SO^2−^_4_ (*R*^2^ = 0.688, *p* < 0.0005, *n* = 16) with increasing isolation age (Figure 6). There were also significant increases in the mass of solute per unit area for DOC (*R*^2^ = 0.405, *p* = 0.008, *n* = 16), DON (*R*^2^ = 0.279, *p* = 0.035, *n* = 16), DIN (*R*^2^ = 0.821, *p* = 0.0005, *n* = 14 with two below detection outliers removed), DOP (*R*^2^ = 0.463, *p* = 0.004, *n* = 16) and PO^3−^_4_ (*R*^2^ = 0.406, *p* = 0.008, *n* = 16) with increasing isolation age of the holes. In contrast, there were no significant (*p* < 0.05) relationships between isolation age and DON:DIN (*R*^2^ = 0.024, *p* = 0.274, *n* = 16), DOP:PO^3−^_4_ (*R*^2^ = 0.001, *p* = 0.913), P_MgCl2_ (*R*^2^ = 0.000, *p* = 0.988), P_NaOH_ (*R*^2^ = 0.007, *p* = 0.773), P_residual_ (*R*^2^ = 0.001, *R*^2^ = 0.929) or N_KCl_ (*R*^2^ = 0.105, *p* = 0.259) (not shown), bacterial production (R^2^ = 0.001, *p* = 0.913) or photosynthesis (*R*^2^ = 0.073, *p* = 0.325) (Figure 6).

### Nutrient mass balance within cryoconite holes

Nutrient fluxes from ice ablation into Canada Glacier cryoconite holes can account for means of 70% of the organic carbon demand, 80% of the nitrogen demand, and 22% of the phosphorus demand of bacterial production (Table 8). Ablation nutrient fluxes account for means of 36% of the dissolved inorganic carbon demand of photosynthesis, 11% of the nitrogen demand and just 2% of the phosphorus demand (Table 8).

**Table 8:** **Estimated nutrient fluxes into *T* = 0 and *T* + 10 cryoconite holes from ice ablation, vs. nutrient demand from bacterial production and photosynthesis (see main text for calculations)**.

	**Ablation flux μmoles m^−2^ h^−1^**	**Bacterial production demand μmoles m^−2^ h^−1^**	**% ablation flux of bacterial production demand**	**Photosynthesis demand μmoles m^−2^ h^−1^**	**% ablation flux of photosynthesis demand**
DOC	0.94	3.5±3.2	70.0±85.7	N/A	N/A
DIC	3.8	N/A	N/A	18.6±14.7	36.4±29.6
TN_(aq)_	0.17	0.54±0.48	79.7±100	2.8±2.2	10.7±8.6
TP_(aq)_	0.0038	0.05±0.04	21.8±27.5	0.23±0.19	2.2±2.4

*Error bars are 1σ (n = 16)*.

We calculate that in holes where potential nitrogen fixation was detected, nitrogen fixation could account for a mean of 12.3 ± 13.3% of the estimated carbon production of photosynthesis, or 119 ± 177% of bacterial production.

## Discussion

### Microbial biogeochemical cycling in entombed cryoconite holes

**Figure 7:**
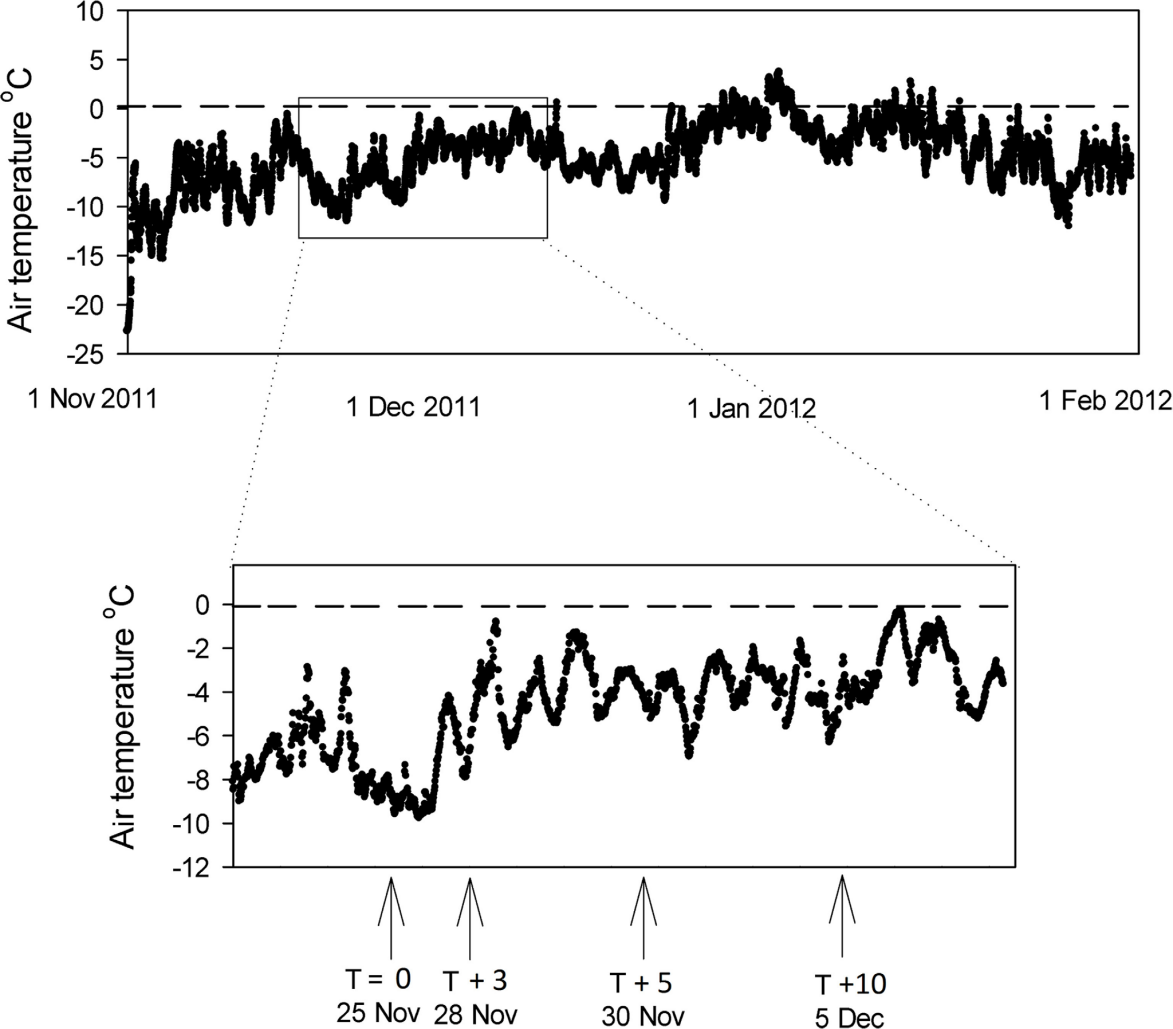
**Air temperature record for the surface of Canada Glacier during the 2011 season**. Data from Fountain, www.mcmlter.org/queries/met/met_stations.jsp#CAAM. Inset shows ice core and cryoconite hole sampling points.

The air temperature above Canada Glacier in the 2011 season is shown in Figure 7 [data from Fountain, www.mcmlter.org/queries/met/met_stations.jsp#CAAM]. The air temperature was consistently below 0°C for the period of this study (mean −4.8°C), and below 0°C for all but a few days of the entire melt season (Figure 7). Despite the subzero temperatures during the study period, the presence of liquid water in holes from *T* + 3 (Table 1) demonstrates that incoming solar radiation through the ice lids was sufficient to warm the cryoconite sediment enough to melt ice (Mueller et al., 2001; Fountain et al., 2004, 2008; Hoffman et al., 2008).

The chemistry of the ice cores and fully frozen cryoconite holes are similar to those described on Canada Glacier in previous melt seasons (Tranter et al., 2004; Bagshaw et al., 2007), and provide evidence for mineral dissolution reactions and active microbial nutrient cycling. The mean isolation age of 3.4 years was within the previously reported ranges of 0–11 years (Fountain et al., 2004; Tranter et al., 2004) and 0–5 years (Bagshaw et al., 2007). Relative to concentrations in surrounding ice, the enrichment of dissolved Ca^2+^, Mg^2+^, and SO^2−^_4_ over Cl^−^ (Table 3) most likely indicates the dissolution of rock debris within the enclosed cryoconite holes (Tranter et al., 2004). The lower mean enrichment factors for aqueous nitrogen species (DON, NO^−^_3_, NO^−^_2_) and phosphorus species (DOP and PO^3−^_4_) relative to Cl^−^ are consistent with the microbiological uptake of nutrients (Tranter et al., 2004).

Our results are consistent with a relationship of increasing aqueous nutrient mass with age (Tranter et al., 2004; Bagshaw et al., 2007). There were significant increases in aqueous inorganic ions (Cl^−^, Na^+^, Ca^2+^, Mg^2+^, SO^2−^_4_, all *p* < 0.0005) and nutrients (TOC, TON, TIN, DOP, and PO^3−^_4_, all *p* < 0.01 other than TON with *p* < 0.05) with increasing isolation age of the holes (Figure 6). While it might be argued that rates of bacterial production and photosynthesis should also increase with greater isolation age due to the higher nutrient load, we found no such significant relationships (*p* > 0.05). It is possible that trends could be masked by the high natural variation in microbial activity rates in individual samples (Figure 6), or by limitation by some other factor (e.g., temperature, or DIC in the case of photosynthesis). Alternatively, it may be that the aqueous concentration of nutrients in the cryoconite hole water column at the time of measurement may be a more important factor than the total mass of nutrients within the cryoconite hole for determining short term microbial activity rates. The impact of freeze-thaw concentration effects on rates of microbial activity is discussed in greater detail in Section Impact of Ionic Pulses on Cryoconite Hole Biogeochemistry.

The cell counts and cell biomass estimates (Table 6) support the presence of a mixed microbial assemblage of both heterotrophs and phototrophs, consistent with previous studies (Christner et al., 2003; Foreman et al., 2007). Autoflourescent cell counts confirmed the presence of a substantial population of phototrophs within the cryoconite sediments (Table 6). The mean measured rates of bacterial production (2.4 ± 1.6 ng C g^−1^ h^−1^, with a maximum of 6.8 ng C g^−1^ h^−1^) were lower than the mean reported for cryoconite holes from a range of MDV glaciers (23.4 ± 11.8 ng C g^−1^ h^−1^; Canada Glacier, Commonwealth Glacier, and Taylor Glacier) (Anesio et al., 2010). This may reflect the higher organic matter contents of cryoconite on Commonwealth Glacier relative to Canada Glacier (Bagshaw et al., 2013). The mean rate of photosynthesis in cryoconite in this study (15.3 ± 11.7 ng C g^−1^ h^−1^, with a maximum of 48 ng C g^−1^ h^−1^) are similar to those reported previously from Canada Glacier using the ^14^C method of 17–58 ng C g^−1^ h^−1^ (reported in Hodson et al., 2013) and 34 ng C g^−1^ h^−1^ using a ΔO_2_ method (Bagshaw et al., 2011), although 4× lower than mean rates in entombed cryoconite holes at Vestfold Hills, Antarctica (92 ± 63 ng C g^−1^ h^−1^) (Hodson et al., 2013).

If we assume that the disparity between the mean organic carbon content of MDV valley floor soil (0.2 mg C g^−1^; Barrett et al., 2007) and Canada Glacier cryoconite (0.9 mg C g^−1^; Table 5) is attributable to net photosynthesis (Bagshaw et al., 2013), then the measured rates of photosynthesis can be used to place some constraints on the possible residence time of cryoconite debris on the surface of the glacier. This seems a reasonable assumption, given that aeolian organic matter derived from the valley floor and deposited onto the glaciers has a lower rather than higher organic matter content than valley soils (Sabacka et al., 2012). The amount of organic matter that would be produced by the mean and maximum rates of photosynthesis rates from this study were 15.3 ng C g^−1^ h^−1^ and 47.8 ng C g^−1^ h^−1^ respectively, equivalent to 26 μg C g^−1^ and 80 μg C g^−1^ over a 10 week melt season. To make up the 700 μg C g^−1^ deficit between MDV soils and Canada glacier cryoconite would require 27 years and 9 years, respectively at these mean and maximum rates of photosynthesis, greater than the 3.4 year mean isolation age of the cryoconite holes. In reality, rates of net autochthonous organic accumulation will be substantially lower than measurements of gross photosynthesis in cryoconite holes, as respiration is likely to recycle a significant fraction of the autotrophic carbon (Telling et al., 2012a; Hodson et al., 2013). Assuming that the rates of photosynthesis measured in this study are representative, the relatively high organic content of the cryoconite could be explained by the cryoconite being trapped within holes on the glacier for several decades, far greater than the average residence time of water within the cryoconite holes. The longer residence time of cryoconite debris over cryoconite water on the glaciers provides a potential explanation for the lack of any trends between cryoconite debris nutrient concentration and the isolation age of the holes (Section Biogeochemistry as a Function of Isolation Age). The cryoconite debris nutrient concentrations may instead reflect the sum of their history in contact with water of a variety of different isolation ages.

Mass balance estimates indicate that nutrient fluxes (C, N, P) from ice ablation alone are unlikely to be able to sustain the measured rates of microbial activity, even with efficient recycling between the heterotrophic and autotrophic communities (Table 8). Particulate inorganic and organic matter in cryoconite debris can provide additional sources of nutrients to cryoconite ecosystems that can help make up the deficit from ice melt (Foreman et al., 2007; Stibal et al., 2008, 2009). While concentrations of loosely adsorbed phosphorus (P_MgCl2_) were close to the detection limit (Table 5) consistent with previous studies on the glacier (Bagshaw et al., 2013) there were higher concentrations of potentially bioavailable inorganic P_NaOH_ (mean of 10.3 ± 3.4 μg P g^−1^). The majority of phosphorus in cryoconite was in the form of P_residual_ (mean of 1455 ± 241 μg P g^−1^), of which approximately 40% may be in the form of organic phosphorus (Bagshaw et al., 2013). The microbial uptake of phosphorus from organic matter in Canada Glacier cryoconite holes is consistent with the previous detection of phosphatase enzyme activity (Foreman et al., 2007). Additional DIC can derive from carbonate dissolution and organic matter remineralization (Tranter et al., 2004). This is consistent with entombed cryoconite hole biogeochemistry at a blue ice Antarctic site (Vestfold Hills) where rates of DIC inputs from ice ablation into entombed cryoconite holes were insufficient to account for all of the measured rates of net ecosystem production (Hodson et al., 2013).

The detection of potential nitrogen fixation in fully melted cryoconite holes (*T* = 0 and *T* + 10; Table 7) suggests that the combination of ice ablation and the recycling of allochthonous organic matter was not always sufficient to meet the full nitrogen requirements of microbial growth. Nitrogen fixation may make up the deficit and ensure that dissolved DOC:TN ratios remain relatively close to those of the Redfield ratio (Figure 3). The rates of areal potential nitrogen fixation measured in the ice lidded cryoconite holes (<1.0–7.9μmoles C_2_H_4_ m^−2^ d^−1^) were similar to those measured in open cryoconite holes on a Greenland glacier of <4.2–16.3 μmoles C_2_H_4_ m^−2^ d^−1^ (Telling et al., 2012b) although lower than the maximum rates measured on Svalbard valley glaciers of 100 μmoles C_2_H_4_ m^−2^ d^−1^ (Telling et al., 2011). Nitrogen fixation could therefore be a significant additional source of nitrogen for microbial growth during the main melt season away from more nitrogen rich ionic pulses (see below), where it could potentially account for 100% of the nitrogen demand of bacterial production and 12% of the nitrogen demand of photosynthesis (Section Nutrient Mass Balance within Cryoconite Holes). When cryoconite is periodically flushed downstream during warmer high melt events (Fountain et al., 2004; Foreman et al., 2007; Bagshaw et al., 2013) nitrogen derived from supraglacial nitrogen fixation will contribute to nutrient fluxes to proglacial streams and perennially ice covered lakes.

### Impact of ionic pulses on cryoconite hole biogeochemistry

Relative to frozen cryoconite holes at *T* = 0, the increase in mean Cl^−^ concentrations >9× during the initial melting (*T* + 3) of cryoconite holes coincident with more negative δ^18^O-H_2_O (Table 2) is consistent with an ionic pulse; the concentration of ions due to the initial melting of brine ice created during freeze up the previous melt season (Fountain et al., 2008). The mean enrichment factors for different ions varied during the ionic pulse (Figure 1), ranging from 0.5× for DIC to 10.2× for Mg^2+^. One possible explanation for the variation in the enrichment factors for different ions could be that they have different partition coefficients from/into the ice crystal structure during freeze-thaw events. The relative order of ion enrichment in the cryoconite hole ionic pulse (Table 3) is however different to that reported for early snow melt (Cragin et al., 1996). For example, in the early melt cryoconite holes (*T* + 3) Cl^−^ has the second largest enrichment factor after Mg^2+^ (excluding C, N, and P; see below) (Table 3) whereas Cl^−^ has the lowest enrichment factor in early snow melt (Cragin et al., 1996). The reason for these differences is unclear and deserves further research. Potential explanations include differences in freezing rates (e.g., a higher freezing rate could result in lower fractionation factors) or variations in solute concentration/speciation. In addition, mineral precipitation reactions may influence the concentrations of ions such as Ca^2+^ and DIC (see below), while microbial activity could influence the concentrations of C, N, and P species.

The behavior of the macronutrients C, N, and P during the ionic pulse is important as they could directly impact microbial activity. The mean enrichment factors for DOC (9.6×), NH^+^_4_ (9.7×), and NO^−^_2_ (9.7×) were similar to Cl^−^ (9.3×) (Figure 1), suggesting a dominant control by freeze-thaw processes. The lower mean enrichment factor for DON (5.6×) and higher enrichment factor for NO^−^_3_ (12.2×) (Figure 1) suggest a short term perturbation of the ecosystem with the temporary increased microbial utilization of dissolved organic nitrogen and nitrification during the ionic pulse. The lack of detectable nitrogen fixation during the ionic pulse (*T* + 3 and *T* + 5; Table 7) is likely due to the relatively high concentrations of both dissolved and cryoconite bound nitrogen species during the ionic pulse event (Figure 4, Table 5), analogous to the inhibition of nitrogen fixation by relatively nitrogen rich snow melt in open cryoconite holes in the Arctic (Telling et al., 2011).

The ionic pulse was associated with significantly (*p* < 0.05) increased rates of bacterial production (Figure 1), alongside a substantial increase in cryoconite bound NH^+^_4__KCl_(Table 5) indicative of increased organic matter remineralization. The enrichment factors for PO^3−^_4_ (1.1×) and DOP (4.4×) during the ionic pulse were lower than for DOC and dissolved nitrogen species, resulting in higher TOC:TP_(aq)_ and TN:TP_(aq)_ratios (Figure 3). The dissolved nutrient stoichiometry suggests that the primary explanation for the elevated rates of bacterial production during the ionic pulse may be an alleviation of phosphorus limitation, with the preferential uptake of PO^3−^_4_ over DOP (Figure 1). The lack of nitrogen fixation during the ionic pulse (Table 7) could potentially contribute to the elevated rates of bacterial production as nitrogen fixation is typically energetically expensive relative to the uptake of dissolved nitrogen (Gutschick, 1978).

Unlike bacterial production, rates of potential photosynthesis were not significantly (*p* > 0.05) elevated during the ionic pulse (Figure 1). Within cryoconite holes, a contributing factor could be light limitation due to shading by either the ice and lid surrounding the hole (Hodson et al., 2013) or by cryoconite grains. While no attempt was made in this study to replicate the exact light levels within the *in situ* cryoconite holes, previous studies on Arctic cryoconite have demonstrated significant self-shading by cryoconite grains in sediment > 1 mm thick (Cook et al., 2010; Telling et al., 2012a). Given that the mean sediment thickness in the Canada glacier cryoconite holes and incubations was >5 mm (Table 1), it seems highly likely that at least some degree of light limitation was present *in situ* if sediment depth integrated rates of photosynthesis are considered. The ultimate limiting factor for photosynthesis in these entombed cryoconite holes may however be DIC. In contrast to all other measured dissolved species, there was a mean net depletion (0.5×) rather than enrichment in mean DIC concentrations during the ionic pulse event (Table 3, Figure 1). Although DIC was measured on only three cryoconite holes during the ionic pulse event due to low water levels in the holes at this time, the similarity of all three measurements suggests that this is a real geochemical feature (Figure 2). The available data is consistent with inorganic carbon limitation of photosynthesis within the cryoconite holes. The CO_2(aq)_ concentrations of this study (*T* + 10 only) ranged from 0.05 to 12.9μmoles L^−1^, with a mean of 2.6 μmoles L^−1^. Freshwater phototrophs typically have a higher affinity for CO_2_ than HCO^−^_3_ (Hein, 1997) with many species unable to make use of HCO^−^_3_ directly (Maberly and Spence, 1983). In a review of the inorganic carbon uptake kinetics of 25 freshwater species of phytoplankton, K_1/2_ values ranged from 0.1 to 170 μmoles L^−1^ CO_2(aq)_, with a median of 13 μmoles L^−1^ (Hein, 1997). This median value is higher than all values of CO_2(aq)_ in cryoconite holes at T+10. There is also a weak but significant correlation (*p* < 0.05) between DIC and rates of photosynthesis (Figure 5), although this correlation is not significant if the single highest rate of photosynthesis is removed. Inorganic carbon limitation of photosynthesis within the entombed cryoconite holes therefore appears plausible, but further research is needed to quantify the DIC uptake kinetics of photosynthetic microbes within the cryoconite holes.

The likely reason for the relatively low DIC concentrations during the ionic pulse is the supersaturation of CaCO_3_ minerals (aragonite or calcite), driving DIC (and Ca^2+^) concentrations down (Tranter et al., 2004). This would be consistent with the relatively low mean enrichment factor for Ca^2+^ during *T* + 3 (2.9× compared to 10.2× for Mg^2+^, and 9.3× for Cl^−^; Figure 1). This supersaturation could occur purely abiotically, via elevated Ca^2+^ and DIC concentrations in an early melt phase prior to measurements at *T* + 3. In addition, elevated nutrient concentrations (DIC, nitrogen, phosphorus) may have promoted higher rates of photosynthesis in an early more nutrient enriched melt water, both consuming DIC and producing alkalinity to further drive carbonate equilibria toward carbonate precipitation (Tranter et al., 2004). This would be consistent with an elevated mean estimated autotrophic cell biomass during *T* + 3 (14.4 μg C g^−1^, relative to 0.9–6.4 μg C g^−1^ at other time points; Table 6), suggestive of a short-lived pulse of higher photosynthetic growth prior to *T* + 3.

Ionic pulses in entombed cryoconite holes are likely important in shifting the timing and maxima of microbial activities to early, and potentially late, in the melt season. It follows that any net increase in nutrient uptake during the ionic pulse would have to be balanced by lower than initial concentrations of nutrients after the ionic pulse events, perhaps reducing rates of microbial activity. This hypothesis is consistent with some data from this study. Concentrations of NH^+^_4_ and NO^−^_2_ are reduced to below detection by the final time point (Table 2) perhaps contributing to the diminished rates of bacterial production (mean of 0.6× of initial levels; Table 7). Mineral precipitation reactions during ionic pulses could further reduce concentrations of nutrients such as DIC to levels that could limit subsequent microbial growth.

### Potential impact of ionic pulses on microbial activity in other cryosphere habitats

The significant enrichment of nutrients and rates of bacterial production by ionic pulses in entombed cryoconite holes has relevance to the ecology of other habitats in the cryosphere where freeze-thaw occurs. Brines contained within veins in ice or permafrost at subzero temperatures are one potential habitat where ionic enrichment through freezing could support increased rates of microbial activity (Rohde and Price, 2007; Barletta et al., 2012). A recent study has suggested that the Antarctic ice sheet could contain >500 km^3^ of brine filled ice veins, potentially supporting viable microbial life (Barletta et al., 2012). The ionic enrichment in veins may facilitate the maintenance and growth of microbial cells in the veins (Price, 2000). Rates of growth are however likely to be constrained by the physical dimensions of the veins, and the length of time that microbes can survive ultimately limited by the depletion of redox couples and nutrients.

A second habitat where freeze thaw events and ionic pulses may have a major impact on microbial activity is the subglacial environment. Under cold based and polythermal glaciers and ice sheets, regelation (pressure melting) of basal ice forms thin films of water, perhaps tens of microns thick (Raiswell et al., 2009), which will likely be enriched in nutrients relative to bulk ice in a similar manner to the cryoconite holes of this study. Nutrient enrichment during regelation is likely to enhance rates of microbial growth; a recent study demonstrated that rates of potential bacterial production (incubations at 2°C) in a MDV glacier (Taylor Glacier) were significantly enhanced by the addition of nutrients (Montross et al., 2013). While microbial growth in regelation films would remove some of these nutrients from solution, additional nutrients could potentially be provided from upstream by the lateral flow of non-regelated ice to the pressure point where pressure melting occurs.

### Conflict of interest statement

The authors declare that the research was conducted in the absence of any commercial or financial relationships that could be construed as a potential conflict of interest.
